# The Honeybee Venom Major Allergen Api m 10 (Icarapin) and Its Role in Diagnostics and Treatment of Hymenoptera Venom Allergy

**DOI:** 10.1007/s11882-020-00943-3

**Published:** 2020-06-16

**Authors:** Thilo Jakob, Michèle Myriam Rauber, Amilcar Perez-Riverol, Edzard Spillner, Simon Blank

**Affiliations:** 1 Department of Dermatology and Allergology, Experimental Dermatology and Allergy Research Group, Justus-Liebig-University Gießen, Gießen, Germany; 2 Department of Engineering Immunological Biotechnology, Aarhus University, Aarhus, Denmark; 3 Center of Allergy and Environment (ZAUM), Technical University of Munich, School of Medicine and Helmholtz Center Munich, German Research Center for Environmental Health, Member of the German Center of Lung Research (DZL), Munich, Germany

**Keywords:** Allergen-specific immunotherapy, Api m 10, Component-resolved allergy diagnostics, Honeybee venom allergy, Icarapin, Venom immunotherapy

## Abstract

**Purpose of Review:**

In Hymenoptera venom allergy, the research focus has moved from whole venoms to individual allergenic molecules. Api m 10 (icarapin) has been described as a major allergen of honeybee venom (HBV) with potentially high relevance for diagnostics and therapy of venom allergy. Here, we review recent studies on Api m 10 characteristics as well as its role in component-resolved diagnostics and potential implications for venom-specific immunotherapy (VIT).

**Recent Findings:**

Api m 10 is a major allergen of low abundance in HBV. It is an obviously unstable protein of unknown function that exhibits homologs in other insect species. Despite its low abundance in HBV, 35 to 72% of HBV-allergic patients show relevant sensitization to this allergen. Api m 10 is a marker allergen for HBV sensitization, which in many cases can help to identify primary sensitization to HBV and, hence, to discriminate between genuine sensitization and cross-reactivity. Moreover, Api m 10 might support personalized risk stratification in VIT, as dominant sensitization to Api m 10 has been identified as risk factor for treatment failure. This might be of particular importance since Api m 10 is strongly underrepresented in some therapeutic preparations commonly used for VIT.

**Summary:**

Although the role of Api m 10 in HBV allergy and tolerance induction during VIT is not fully understood, it certainly is a useful tool to unravel primary sensitization and individual sensitization profiles in component-resolved diagnostics (CRD). Moreover, a potential of Api m 10 to contribute to personalized treatment strategies in HBV allergy is emerging.

## Introduction

Allergic reactions to stings of honeybees may represent severe and even fatal scenarios. Honeybees belong to the order Hymenoptera, which also includes other allergy-relevant species such as yellow jackets, paper wasps, hornets, bumblebees, or stinging ants. Hymenoptera venom allergy can be effectively cured by venom-specific immunotherapy (VIT), provided that the culprit insect can be correctly identified. VIT with honeybee venom (HBV) is reported to be effective in 77 to 84% of HBV-allergic patients and, therefore, is less effective as VIT with vespid venom (91–96%) [1]. Reasons for this discrepancy are so far not clear and a matter of debate.

HBV comprises a variety of different bioactive compounds such as low molecular weight molecules, peptides, and proteins. A honeybee sting contains approximately 59 (± 7) μg of proteins/peptides [2], which are the main triggers of allergic reactions to the venom. In the last two decades, the evolvement of genomic and proteomic approaches has led to the identification of a variety of formerly unknown HBV proteins [3, 4] and shifted the view from the whole venom to individual allergenic molecules [5].

Detailed component-resolved analyses of specific IgE (sIgE) sensitization of HBV-allergic patients using recombinant forms of newly described apian venom proteins elucidated that HBV contains more relevant major allergens than formerly anticipated [6•]. Additionally, these analyses demonstrated that not only venom proteins of high abundance, such as the well-characterized phospholipase A2 (Api m 1), but also proteins that make up only minute amounts of the venom, may represent major sIgE-sensitizing components.

One of these allergens of low abundance is Api m 10, alias icarapin, that in recent years has evoked many research efforts and speculations about its relevance in the diagnosis and therapy of HBV allergy. Api m 10 was first identified by Peiren et al. [3] after a 2D-SDS-PAGE separation of pure HBV (*Apis mellifera*) followed by mass spectrometry analysis of excised spots and was initially referred to as “new venom protein 2.” In the same year, this 204 amino acid (aa), carbohydrate-rich protein with 2-4 potential N-linked glycosylation sites was also described by another group using reversed phase chromatography and IgE binding was reported for the first time [7]. In the following year, Api m 10 was produced as insoluble protein in *E. coli* and IgE binding to the recombinant protein was confirmed [8]. In addition, they were able to localize Api m 10 in the cuticular lining of the venom duct of the honeybee with a minor signal in secretory cells. Furthermore, Api m 10 was described as instable and of low abundance in HBV. The proposed name icarapin is an artificial term combining “Icarus” from the Greek mythology and the genus’ name “Apis” and indicates its instable nature and rapid degradation.

In 2011, Api m 10 was produced for the first time as soluble recombinant protein in *E. coli* and eukaryotic insect cells [9••]. Pronounced IgE reactivity of the proteins was demonstrated in larger cohorts of beekeepers and HBV-allergic patients by Blank et al. [9••]. Moreover, its capability to activate effector cells from HBV-allergic patients was shown in basophil activation test (BAT). At the same time, Api m 10 was listed as HBV allergen in the official allergen nomenclature database of the World Health Organization and International Union of Immunological Societies (WHO/IUIS) [10]. Within HBV, Api m 10 has a share of less than 1% of the dry weight [9••], which is a relatively low amount compared with other allergens such as Api m 1 (12%) or Api m 4 (50%) [5]. Nevertheless, Api m 10 represents a major HBV allergen [6•, 9••] with high relevance for diagnostic approaches [11•, 12•, 13•]. Moreover, it is of potential clinical relevance, as a dominant sensitization to Api m 10 prior to the initiation of VIT has been associated with a higher risk of treatment failure during VIT [14••].

### Api m 10 Homologs and Isoforms

**Fig. 1:**
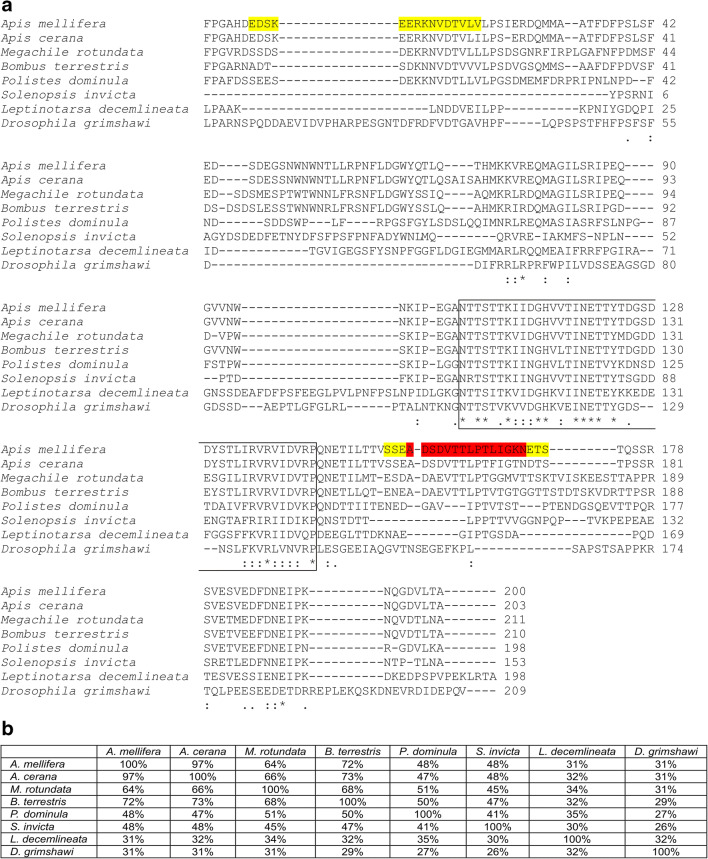
Homologs of Api m 10. **a** Alignment of the mature sequences of *Apis mellifera* Api m 10 variant 2 and homologous proteins from other insect species. The box indicates the conserved region found in all icarapin-like proteins. Asterisks, colons, and periods indicate identical, conserved, and semi-conserved residues, respectively. IgE epitopes that are recognized by more than 40% and 100% of Api m 10-sensitized patients are indicated in yellow and red, respectively. **b** Percent identity between icarapin-like proteins of different insect species. Sequence identifiers: *A. mellifera* (AHM25029.1), *A. cerana* (NP_001315405.1) *M. rotundata* (XP_003704678.2), *B. terrestris* (XP_003396228.1), *P. dominula* (XP_015185877.1), *S. invicta* (XP_011166768.1), *L. decemlineata* (XP_023013082.1), *D. grimshawi* (XP_001989292.1)

Api m 10 is a protein of so far unknown function and contains no known functional domains. Nevertheless, it is a conserved protein, as icarapin-like proteins were identified in various species of the phylogenic class *Insecta*. These include many members of the order Hymenoptera such as bees (e.g., *Apis cerana* and the leafcutting bee *Megachile rotundata*) and bumblebees (e.g., *Bombus terrestris*), wasps (e.g., *Polistes dominula*) and ants (e.g., *Solenopsis invicta*), but also beetles (e.g., *Leptinotarsa decemlineata*), flies (e.g., *Drosophila grimshawi*), fleas, mosquitos, termites, thrips, and bugs. Selected Api m 10 homologs are shown in Fig. 1a. As not all of these insects are venomous, icarapin-like proteins might have evolved to different functions in the different phyla of insects. Although icarapin-like proteins of different species show a wide range of sequence identity (Fig. 1b), all of them share a consensus sequence of approximately 37 to 41 residues (Fig. 1a). Interestingly, Api m 10 as well as all of its homologs do not contain any cysteine residues. Almost all identified Api m 10 homologs from other species are predicted proteins, derived from genomic sequences. Therefore, it is not certain if these genes are really expressed in all species or in which tissues the protein products might be present. The only other organism, beside the honeybee *Apis mellifera*, in which a Api m 10 homolog was identified as a venom component on proteomic level is the European paper wasp *Polistes dominula* [15].

**Fig. 2:**
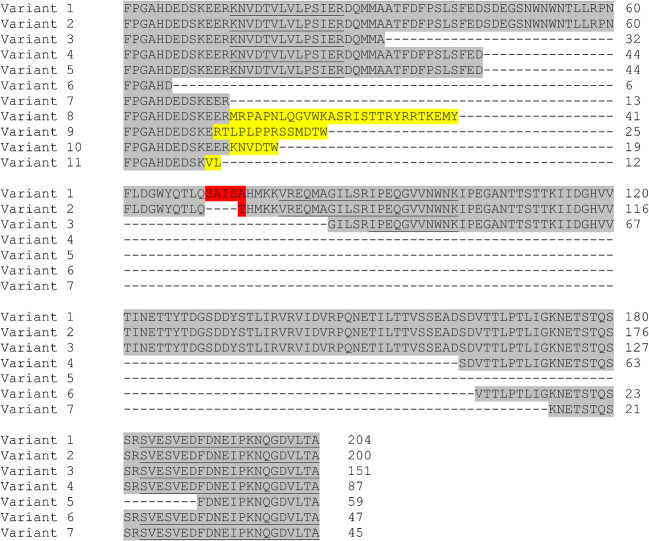
Api m 10 variants. Variants of *Apis mellifera* Api m 10 that were identified on proteomic or transcriptomic level. Highlighted in gray are regions coded by the primary genomic sequence. Shown in red are differences between variants 1 and 2 resulting from alternative splicing. Yellow indicates unique sequences resulting from frame shifts and premature stop codons in chimeric transcripts. Underlined are peptides identified by mass spectrometry of HBV in different studies [3, 4, 9••, 18]. So far, no proteomic evidence exists for the presence of variants 3 to 11 in HBV

Various transcript variants of Api m 10 are described that are coded by one single gene that consists of 4 exons [8, 16]. The Api m 10 variants 1 (204 aa) and 2 (200 aa) are generated by alternative splicing following the general canonical GT-AG splicing rule [17] at the end of exon 2 due to the presence of an alternative splice acceptor site in exon 3 [8, 16]. The two isoforms differ by only 12 base pairs (bb) resulting in an exchange from SAISA to T in variant 2 (Fig. 2), which is listed in the WHO/IUIS allergen nomenclature database. All other 9 shorter Api m 10 isoforms are a result of so-called chimeric transcripts that are generated by joining exon 2 to exon 3 or 4 from different transcripts at short homologous sequences (SHSs) present in the exons [16]. The variants 3 to 7 (151, 87, 59, 47, and 45 aa) are fragments consisting of amino acid sequences also present in variants 1 and 2, while the genetic process in the very short variants 8 to 11 (41, 25, 19, and 12 aa) leads to frame shifts and, hence, unique isoform-specific sequences and premature stop codons (Fig. 2). Most of these unique isoforms did not display IgE reactivity, whereas the isoforms more similar to variants 1 and 2 displayed the highest IgE reactivity [16]. While the presence of variants 1 and/or 2 in the venom was confirmed by the identification of a variant-specific peptide, the existence of all other isoforms at protein level could not be proven by the detection of isoform-specific peptides (Fig. 2) [3, 4, 9••]. The observation that Western blot analyses of HBV preparations with monoclonal and polyclonal Api m 10-specific antibodies only detect reactivity in a molecular weight range corresponding to variants 1 and 2 [9••, 14••, 19••] seems to suggest that the described chimeric transcripts may not be translated into protein in vivo.

### Structural Aspects of Api m 10

**Fig. 3:**
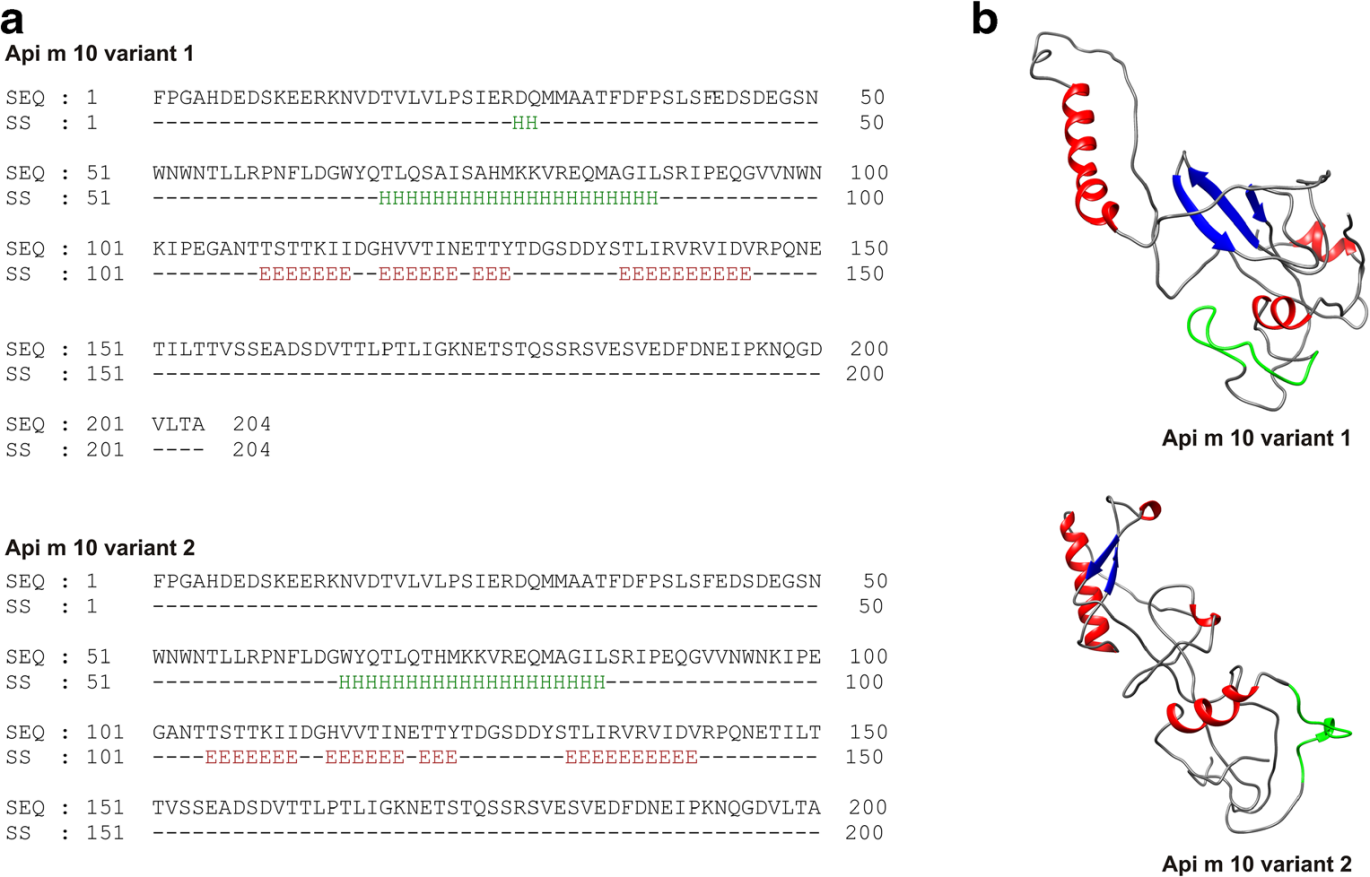
Api m 10 structure. **a** Predicted secondary structure of Api m 10 variants 1 and 2. H and E indicate α-helical and β-stranded regions, respectively. Other regions are predicted to be unstructured (coiled). **b** Predicted structural models of Api m 10 variants 1 and 2. α-helices, β-strands, and coiled regions are shown in red, blue, and gray, respectively. The dominant IgE epitope of Api m 10 [21•] is shown in green

Secondary structure predictions for Api m 10 variants 1 and 2 (SPIDER3 [20]) reveal the presence of large unstructured regions. Only one α-helical region in the area where both variants differ is predicted with high probability. Moreover, short β-stranded regions are predicted (Fig. 3a). The 3-dimensional structure of Api m 10 has not been solved so far. Its largely unfolded nature also leads to predicted protein models (PHYRE^2^ [22]) that are primarily unstructured (Fig. 3B). Of note, as the major part of the Api m 10 sequence is likely to be disordered, these regions cannot be meaningfully predicted and resulting models are characterized by a certain degree of unreliability. Thus, the probable flexibility of the unstructured regions may lead to different tertiary structures. Nevertheless, both models contain the α-helical region shown in the secondary structure prediction, whereas, in contrast to the model of variant 2, the Api m 10 variant 1 model also mainly corresponds to the secondary structure prediction in β-stranded regions. Certainly, the flexible structure of Api m 10 is in part due to the absence of any cysteine residues and, hence, disulphide bridges. The unstructured nature of Api m 10 is additionally supported by preliminary 1D proton NMR experiments done with Api m 10, recombinantly produced in bacteria and insect cells (unpublished data). The apparent lack of a defined structure of Api m 10 makes it even more difficult to speculate about putative functions in the venom. Possibly, binding of a ligand/protein partner in the venom or after injection of the venom may induce structural changes (induced fit) or a specific structure may be arranged by multimerisation.

### IgE Sensitization to Api m 10

IgE reactivity to Api m 10 was shown in 2006 for 4 out of 5 HBV-allergic beekeepers [8]. Api m 10 sensitization in a larger patient cohort was first assessed in 2011 by Blank et al. using recombinant Api m 10 devoid of cross-reactive carbohydrate determinants (CCDs) in ELISA [9••]. Here, 53% (27/51) of patients sensitized to HBV and yellow jacket venom (YJV) and 47% (8/17) of patients monosensitized to HBV (52% of all patients) exhibited sIgE to Api m 10, respectively. None of the patients monosensitized to YJV (*n* = 16) displayed any sIgE reactivity to Api m 10. Following these results, Api m 10 sIgE diagnostic started to evolve and first sensitization data using experimental CCD-free Api m 10 sIgE assays at the ImmunoCAP™ platform (Thermo Fisher Scientific, Uppsala, Sweden), which is more standardized and more applicable for measurements in larger study cohorts, was published by Köhler et al. [6•]. In this study, sensitization to Api m 10 (≥ 0.35 kU_A_/L) was detected in 62% (89/144) of HBV-allergic patients. Also, here, no relevant IgE reactivity was measured in patients allergic to YJV (*n* = 40). Interestingly, patients double-sensitized to HBV and YJV exhibited a higher rate of sensitization to Api m 10 (73%, 66/90) compared with HBV-monosensitized patients (43%, 23/54). This was also true for all other tested venom allergens. Moreover, double-sensitized patients mostly showed higher levels of sIgE to whole HBV as well as to HBV allergens compared with monosensitized patients, suggesting that this might be due to a more advanced state of allergic immune deviation in the double-sensitized population [13•]. In a follow-up study assessing component-resolved allergen sensitization in double-sensitized Hymenoptera venom-allergic patients who were negative for the HBV major allergen Api m 1, Api m 10 sensitization was detected in 47% and 43% of patients for whom the culprit insect was either unknown or identified as honeybee, respectively [23••].

In 2015, Api m 10 became commercially available (ImmunoCAP™) for routine diagnostics. Shortly after, Frick et al. [14••] retrospectively analyzed Api m 10 sensitization in a patient cohort before the initiation of HBV VIT and found Api m 10 sIgE (≥ 0.35 kU_A_/L) in 72% (83/115) of patients. Of note, in this study, the patient population was composed of two thirds of patients who successfully underwent VIT and of one third for whom therapy failed according to sting challenge tests. Hence, the Api m 10 sensitization rate might be biased by the patient selection. Considerably lower sensitization rates were described in an Austrian study by Arzt et al. [24]. Here, Api m 10 sensitization was reported for 35% in both groups, HBV-monosensitized patients (47/134) and HBV and YJV double-sensitized patients (19/55). Interestingly, a higher Api m 10 sensitization rate in double-sensitized patients was not found in this study, although this was the case for other allergens such as Api m 1, Api m 3, and Api m 5. Vachova et al. reported sensitization to Api m 10 in 55% (60/110) of HBV-allergic patients (47% (28/29) and 63% (32/51) in HBV-monosensitized and HBV/YJV double-sensitized patients, respectively) [25]. An overview of Api m 10 sensitization rates in different studies is given in Table 1. As patient inclusion criteria and geographical differences in sensitization profiles certainly influence study outcome, large international multicenter studies are needed to assess the relevance of particular allergens in detail.

**Table 1:** Rates of sensitization to Api m 10 in different study cohorts

Number of patients	Patient characteristics	Api m 10 sIgE positive	Method	Reference
68	HBV-sensitized	35 (52%)	ELISA	Blank et al. 2011 [9••]
51	HBV /YJV ds	27 (53%)		
17	HBV ms	8 (47%)		
144	HBV-sensitized	89 (62%)	ImmunoCAP^*^	Köhler et al. 2014 [6•]
90	HBV/YJV ds	66 (73%)		
54	HBV ms	23 (43%)		
58	HBV/YJV ds Api m 1 sIgE neg. Culprit insect unknown	27 (47%)	ImmunoCAP^*^	Frick et al. 2015 [23••]
28	HBV/YJV ds Api m 1 sIgE neg. Culprit insect honeybee	12 (43%)		
115	HBV-sensitized^**^	83 (72%)	ImmunoCAP^*^	Frick et al. 2016 [14••]
189	HBV-sensitized	66 (35%)	ImmunoCAP^*^	Arzt et al. 2017 [24]
55	HBV/YJV ds	19 (35%)		
134	HBV ms	47 (35%)		
110	HBV-sensitized	60 (55%)	ImmunoCAP^*^	Vachova et al. 2018 [25]
51	HBV/YJV ds	32 (63%)		
59	HBV ms	28 (47%)		

HBV-sensitized patient populations include HBV-monosensitized and HBV/YJV double-sensitized patients

*ds* double-sensitized, *ms* monosensitized, *neg*. negative, *HBV* honeybee venom, *YJV* yellow jacket venom

^*^Values ≥ 0.35 kU_A_/L were considered positive

^**^In this study, the patient population is composed of two thirds VIT responders and one third non-responders. Hence, the obtained sensitization rate might be biased

The ability of Api m 10 to also induce considerable effector cell activation was confirmed by BAT [9••]. Here, the basophils of 62% (8/13) of consecutively recruited HBV-allergic patients were activated in a dose-dependent manner by Api m 10.

### IgE Epitopes of Api m 10

Very recently, linear IgE epitopes of Api m 10 variant 1 were investigated using a macroarray platform coated with 15-mer peptides (overlap of 12 aa) spanning the whole amino acid sequence of the mature protein [21•]. Here, all tested sera of Api m 10 positive (ImmunoCAP™, sIgE ≥ 0.35 kU_A_/L) HBV-allergic patients (*n* = 40) exhibited sIgE reactivity that showed a reasonable correlation with the values obtained by ImmunoCAP™. Healthy control sera exhibited no reactivity. Sera of individual patients were reactive with up to 29 of the peptides and overall 43 of 64 peptides were recognized. Interestingly, three regions with IgE binding of more than 40% of the sera were identified (Fig. 1a). Of major interest is one peptide (Fig. 1a, red; Fig. 3b, green) that was recognized by 100% of the Api m 10-sensitized patients (Api m 10_160–174_; amino acid sequence: ADSDVTTLPTLIGKN; patent pending registration number EP19199738) and that exhibited higher sIgE reactivity than any other of the peptides (67% of the total Api m 10 peptide sIgE). The concentrations of Api m 10_160–174_ sIgE correlated well with the concentrations of sIgE to full-length Api m 10. Moreover, in ImmunoCAP™ inhibition experiments sIgE binding to full-length Api m 10 could be inhibited up to 80% in a dose-dependent manner by Api m 10_160–174_. Vice versa, preincubation of sera with full-length Api m 10 was able to prevent sIgE binding to Api m 10_160–174_. Taken together, these data suggest the existence of a major IgE epitope of Api m 10 that is of relevance for all tested patients and, hence, might be a potential target for the development of future diagnostic tools and therapeutic applications.

### Role of Api m 10 in Diagnostics of Hymenoptera Venom Allergy

In recent years, CRD of Hymenoptera venom allergy has more and more evolved, particularly for the discrimination between HBV and YJV allergy [11•, 12•, 13•]. In contrast to extract-based sIgE diagnostics, which uses whole venom preparations [26], in CRD, sIgE levels to individual relevant allergens are determined. CRD with recombinant CCD-free allergens has proven high potential for the discrimination between cross-reactivity and true primary sensitization to different venoms [23••, 27–30, 31•] as well as for improving sensitivity of sIgE detection [32]. Particularly, the use of species-specific marker allergens that are present exclusively in HBV or vespid venom has mainly improved identification of primary sensitization to a given venom. The fact that YJV-allergic patients lack IgE reactivity to Api m 10 [6•, 9••] has added Api m 10 to the panel of species-specific marker allergens of HBV that further comprises Api m 1, Api m 3, and Api m 4. The identification of an Api m 10 homolog in *Polistes dominula* venom (PDV) (see above) has raised the question if this marker allergen concept also holds true for the discrimination between HBV and PDV allergy. However, our own preliminary unpublished data suggests missing cross-reactivity between Api m 10 and its homolog in PDV (48% aa sequence identity) and/or a negligible role of PDV icarapin as sensitizing component of PDV. Intriguingly, the major IgE epitope identified in Api m 10 is not present in its PDV homolog (Fig. 1a).

In the early days of CRD of Hymenoptera venom allergy, exclusively, the major allergen Api m 1 was used for diagnosis of HBV allergy. Depending on the assessed patient population and the test system used, the diagnostic sensitivity of this single allergen for the detection of HBV sensitization ranged between 58 and 97% [6•, 27, 29, 33–35]. In the first study that applied an experimental allergen panel for the detection of HBV sensitization (*n* = 144; 54 HBV-monosensitized and 90 HBV/YJV double-sensitized), the addition of Api m 10 to Api m 1 increased diagnostic sensitivity from 72 to 87%, whereas the combination of 6 allergens (Api m 1-5 and 10) resulted in a sensitivity of 94% (sIgE ≥ 0.35 kU_A_/L) [6•]. In another study, using the same assay platform, combining the allergens Api m 1-3, 5, and 10 lead to a diagnostic sensitivity of only 79% [24], most likely due to a different composition of the patient population, in particular the number of HBV-monosensitized (*n* = 134; diagnostic sensitivity 72%) and HBV and YJV double-sensitized patients (*n* = 55; diagnostic sensitivity 93%). For the same allergen panel Vachova et al. reported a diagnostic sensitivity of 92% in the whole population of HBV-allergic patients and of 90% and 94% in HBV-monosensitized and HBV/YJV double-sensitized patients, respectively [25].

Taking into consideration, that CRD is particularly important for the elucidation of double-sensitization, the commercial availability of a panel of marker allergens can be considered highly valuable for adequate diagnostics. This especially holds true for the difficult to manage group of patients who are double-sensitized to HBV and YJV and were not able to identify the culprit insect. Recently, it was demonstrated that in this patient population (*n* = 126), a primary sensitization to HBV could be confirmed in 54% (68) of cases using Api m 1. Interestingly, in the remaining Api m 1 negative patients, sIgE (≥ 0.35 kU_A_/L) to the marker allergens Api m 3 and Api m 10 was found in 29% (15) and 47% (27), respectively. Hence, these two allergens confirmed primary sensitization to HBV in 65% (31) of the Api m 1 negative population [23••]. This is of particular relevance, as without the additional sIgE measurements, those patients would have been regarded as having a sensitization only to YJV and not to HBV. Therefore, sIgE measurements to additional allergens such as Api m 10 and Api m 3 can build a reasonable basis for the detection of primary sensitization and, thus, for the initiation of potentially life-saving VIT. A subsequently published study questioned the ability of the currently available allergen panel to resolve double-sensitization, as 70% (69/98) of HBV and YJV double-sensitized patients were also double-sensitized with at least one recombinant allergen of HBV and YJV (sIgE ≥ 0.35 kU_A_/L) [36•]. The limitation of this study is that Api m 5 was included as marker allergen for HBV sensitization, despite the fact that it is highly cross-reactive with the YJV homolog Ves v 3. Thus, the study does not allow differentiating to which extent this was caused by cross-reactivity or true primary sensitization to both venoms.

### Api m 10 Content of Therapeutic Venom Preparations

As discussed before, Api m 10 is an apparently unstable allergen of low abundance in HBV [8, 9••]. The instable nature of Api m 10 does not only apply to native Api m 10 in HBV but also to the recombinant allergen [8, 19••]. Considering these facts, the Api m 10 content of approved therapeutic HBV preparations used for VIT was assessed in different studies [9••, 14••, 19••, 37]. Applying polyclonal rabbit Api m 10-specific antisera in semi-quantitative immunoblotting approaches, it was demonstrated that in all tested aqueous HBV preparations, Api m 10 was underrepresented in comparison with freshly prepared crude HBV [14••, 19••, 37]. Intriguingly, Api m 10 was undetectable in all tested batches of a highly purified HBV preparation (Aquagen®, ALK-Abelló) by this approach. For the production of this preparation, HBV is further processed to remove biogenic amines and to reduce the content of small peptides. While in three of the less purified HBV preparations a certain amount of Api m 10 was detectable in all tested batches (Pharmalgen®, ALK-Abelló; Venomil®, Allergy Therapeutics; Alyostal Venin®, Stallergenes), a fourth product showed an obvious batch-to-batch variation (Venomenhal®, HAL Allergy). Analyzing aluminum hydroxide-adsorbed depot preparations by such an approach is challenging. However, the most commonly used depot preparation is prepared using the highly purified HBV preparation.

Reasons for these differences between the HBV preparations are so far unclear. Nevertheless, it might be speculated that more intensive HBV processing supports degradation of Api m 10. Although rapid Api m 10 degradation can be slowed down by the addition of human serum albumin, which is contained in the reconstitution solution for HBV VIT preparations, it might be preferable to use freshly reconstituted products for patients with relevant Api m 10 sensitization.

Another study detected Api m 10 peptides in all preparations by mass spectrometry [37], while detection by immunoblotting that covers full-length Api m 10 confirmed the previous results reported by Blank et al. [19••] and Frick et al. [14••]. This apparent discrepancy can be explained by the different methods used. In general, mass spectrometry is usually a non-quantitative approach that detects peptides of proteolytic digests of venom proteins, while immunoblotting allows the detection of full-length protein present in the venom. Subsequent advanced quantitative mass spectrometric analyses confirmed the strongly varying Api m 10 content of the different VIT preparations but also demonstrated significant differences between batches of the same products [38].

Importantly, it is not known whether these differences between HBV preparations may influence the success of VIT, especially in patients with particular sensitization profiles. Prospective studies that would help to answer this question are still lacking. Nevertheless, the detailed knowledge of CRD-based sensitization profiles of patients and of the allergen content of individual HBV preparations paves the way to select the potentially most suitable preparation in a patient-tailored manner.

### Api m 10 and Its Impact on Treatment

Component-resolved analysis of patients’ sensitization profiles might also be helpful for personalized risk stratification in VIT. In 2016, Frick et al. [14••] demonstrated in a retrospective multicenter study of VIT-treated HBV-allergic patients that a predominant sensitization to Api m 10 (defined as > 50% of sIgE to HBV) prior to the initiation of VIT represents a relevant risk factor for treatment failure (according to sting challenge tests) with an odds ratio of 8.44. Such an association was not found for dominant sensitization to other tested allergens such as Api m 1, Api m 2, Api m 3, and Api m 5. Interestingly, all patients who exhibited sIgE to Api m 10 higher than 60% of HBV sIgE were therapy non-responders. As a caveat, it has to be mentioned that this study used a retrospective design with a univariate analysis not considering other identified confounders for VIT failure in a multivariate analysis. Along the same line, Köhler et al. demonstrated that HBV VIT only induced minimal IgG4 to low abundance allergens such as Api m 10 while substantial IgG4 induction to high abundance allergens such as Api m 1 and Api m 4 was observed [6•]. Although IgG4 induction per se is no marker for therapeutic success, this suggests differences in the immunological response or even unresponsiveness to particular underrepresented allergens. Dominant Api m 10 sensitization was reported in 10% (12/115) [14••] to 12% (17/144) [6•] of HBV-allergic patients in populations with high Api m 10 sensitization rates (62–72%), while in a population with a low sensitization rate to Api m 10 (35%) dominant sensitization was reported only in 6% (11/189) [24]. Even though prospective studies are still missing and the role of Api m 10 in HBV allergy and tolerance induction during VIT is not fully understood, the data by Frick et al. suggest that patients with dominant Api m 10 sensitization are at increased risk for treatment failure during honeybee VIT and preferably should be treated with a venom preparation that contains amounts of Api m 10 detectable by Western blot. This has to be taken with a grain of salt, as this assumption is based on an association observed in a single retrospective multicenter study. Currently, we do not know if and how much Api m 10 or any of the other low abundance allergens is required for successful VIT. Prospective studies comparing VIT with HBV preparations of different allergen content would allow addressing this kind of questions.

Nevertheless, how the knowledge of patient sensitization profiles can help to adapt treatment protocols is described in a very recent prospective case report [39•]. Here, a HBV-allergic beekeeper with dominant (near exclusive) Api m 10 sensitization was up-dosed in a cluster schedule to a maintenance dose of 300 μg with a venom preparation for that detectable Api m 10 content was shown before [14••, 19••] and also demonstrated in sIgE inhibition experiments. Monthly injections for 2 years were well tolerated and performed with freshly reconstituted venom preparations to exclude Api m 10 degradation. Controlled sting challenge tests 1 and 2 years after treatment started as well as a field sting were well tolerated. Moreover, induction of Api m 10 sIgG4 was demonstrated. Along the same line, a different case report demonstrated that changing treatment to a less purified HBV preparation can successfully induce tolerance in a patient, in which treatment with 300 μg of a highly purified HBV preparation was not successful [40]. Unfortunately, the authors did not report whether this was associated with a dominant Api m 10 sensitization.

Even though it is not finally resolved that the significantly higher number of therapy failures in HBV VIT compared with vespid venom VIT [1, 41] is causally linked with the low content of particular allergens in HBV or particular sensitization profiles, the evidence obtained so far surely might help to guide towards a personalized precision-targeted VIT.

## Conclusions

The continuously expanding knowledge of the allergen composition of Hymenoptera venoms and the relevance of particular allergens will permit enhanced precision in diagnostics and management of venom-allergic patients. Api m 10 is a major sensitizing allergen of HBV, which has led to an increased sensitivity of CRD and improved dissection of primary sensitization and cross-reactivity in venom allergy. Hence, Api m 10, together with other marker allergens, in many cases facilitates therapeutic decisions and correct prescription of VIT. Moreover, dominant sensitization to Api m 10 represents a relevant risk factor for failure of HBV VIT, possibly due to its underrepresentation in some HBV preparations, commonly used for therapy. Although the role of Api m 10 in tolerance induction during VIT is not completely understood and prospective studies are still missing, the knowledge of patients’ sensitization profiles and given evidence might allow a better risk stratification in VIT and a personalized treatment.

## References

[1] Sturm GJ, Varga EM, Roberts G, Mosbech H, Bilo MB, Akdis CA (2018). EAACI guidelines on allergen immunotherapy: Hymenoptera venom allergy. Allergy..

[2] Hoffman DR, Jacobson RS (1984). Allergens in hymenoptera venom XII: how much protein is in a sting?. Ann Allergy.

[3] Peiren N, Vanrobaeys F, de Graaf DC, Devreese B, Van Beeumen J, Jacobs FJ (2005). The protein composition of honeybee venom reconsidered by a proteomic approach. Biochim Biophys Acta.

[4] Van Vaerenbergh M, Debyser G, Devreese B, de Graaf DC (2014). Exploring the hidden honeybee (Apis mellifera) venom proteome by integrating a combinatorial peptide ligand library approach with FTMS. J Proteome.

[5] Spillner E, Blank S, Jakob T (2014). Hymenoptera allergens: from venom to "venome". Front Immunol.

[6] Köhler J, Blank S, Müller S, Bantleon F, Frick M, Huss-Marp J (2014). Component resolution reveals additional major allergens in patients with honeybee venom allergy. J Allergy Clin Immunol.

[7] Schmidt MWE, Sakell RH, Hoffman DR (2005). Proteins in the high molecular weight fraction of honeybee venom. J Allergy Clin Immunol.

[8] Peiren N, de Graaf DC, Brunain M, Bridts CH, Ebo DG, Stevens WJ, Jacobs FJ (2006). Molecular cloning and expression of icarapin, a novel IgE-binding bee venom protein. FEBS Lett.

[9] Blank S, Seismann H, Michel Y, McIntyre M, Cifuentes L, Braren I (2011). Api m 10, a genuine *A. mellifera* venom allergen, is clinically relevant but underrepresented in therapeutic extracts. Allergy.

[10] Radauer C, Nandy A, Ferreira F, Goodman RE, Larsen JN, Lidholm J, Pomés A, Raulf-Heimsoth M, Rozynek P, Thomas WR, Breiteneder H (2014). Update of the WHO/IUIS allergen nomenclature database based on analysis of allergen sequences. Allergy..

[11] Bilo MB, Ollert M, Blank S (2019). The role of component-resolved diagnosis in Hymenoptera venom allergy. Curr Opin Allergy Clin Immunol.

[12] Blank S, Bilo MB, Ollert M (2018). Component-resolved diagnostics to direct in venom immunotherapy: important steps towards precision medicine. Clin Exp Allergy.

[13] Jakob T, Müller U, Helbling A, Spillner E (2017). Component resolved diagnostics for hymenoptera venom allergy. Curr Opin Allergy Clin Immunol.

[14] Frick M, Fischer J, Helbling A, Rueff F, Wieczorek D, Ollert M (2016). Predominant Api m 10 sensitization as risk factor for treatment failure in honey bee venom immunotherapy. J Allergy Clin Immunol.

[15] Grosch J, Hilger C, Bilò MB, Kler S, Schiener M, Dittmar G, Bernardin F, Lesur A, Ollert M, Schmidt-Weber CB, Blank S. Shedding light on the venom proteomes of the allergy-relevant Hymenoptera Polistes dominula (European paper wasp) and Vespula spp. (yellow jacket) Toxins 2020;12(5):E323. 10.3390/toxins12050323 PMC7291082 32422898

[16] Van Vaerenbergh M, De Smet L, Rafei-Shamsabadi D, Blank S, Spillner E, Ebo DG (2015). IgE recognition of chimeric isoforms of the honeybee (Apis mellifera) venom allergen Api m 10 evaluated by protein array technology. Mol Immunol.

[17] Xiong F, Gao J, Li J, Liu Y, Feng G, Fang W, Chang H, Xie J, Zheng H, Li T, He L (2009). Noncanonical and canonical splice sites: a novel mutation at the rare noncanonical splice-donor cut site (IVS4+1A>G) of SEDL causes variable splicing isoforms in X-linked spondyloepiphyseal dysplasia tarda. Eur J Hum Genet.

[18] Van Vaerenbergh M, Cardoen D, Formesyn EM, Brunain M, Van Driessche G, Blank S (2013). Extending the honey bee venome with the antimicrobial peptide apidaecin and a protein resembling wasp antigen 5. Insect Mol Biol.

[19] Blank S, Etzold S, Darsow U, Schiener M, Eberlein B, Russkamp D (2017). Component-resolved evaluation of the content of major allergens in therapeutic extracts for specific immunotherapy of honeybee venom allergy. Hum Vaccin Immunother.

[20] Heffernan R, Paliwal K, Lyons J, Singh J, Yang Y, Zhou Y (2018). Single-sequence-based prediction of protein secondary structures and solvent accessibility by deep whole-sequence learning. J Comput Chem.

[21] • Rauber MM, Rossbach A, Jung A, Müller S, Moebs C, Pfützner W, et al. The honey bee venom allergen Api m 10 displays one major IgE epitope, Api m 10_160–174_. Allergy. 2020. 10.1111/all.14187. **The study identified one major IgE epitope of Api m 10 with potential for future developments in diagnostics of Hymenoptera venom allergy.**

[22] Kelley LA, Mezulis S, Yates CM, Wass MN, Sternberg MJ (2015). The Phyre2 web portal for protein modeling, prediction and analysis. Nat Protoc.

[23] Frick M, Müller S, Bantleon F, Huss-Marp J, Lidholm J, Spillner E (2015). rApi m 3 and rApi m 10 improve detection of honey bee sensitization in Hymenoptera venom-allergic patients with double sensitization to honey bee and yellow jacket venom. Allergy.

[24] Arzt L, Bokanovic D, Schrautzer C, Schwarz I, Laipold K, Aberer W, Sturm GJ (2017). Questionable diagnostic benefit of the commercially available panel of bee venom components. Allergy..

[25] Vachova M, Panzner P, Kopac P, Bidovec Stojkovic U, Korosec P (2018). Routine clinical utility of honeybee venom allergen components. J Allergy Clin Immunol Pract.

[26] Blank S, Haemmerle S, Jaeger T, Russkamp D, Ring J, Schmidt-Weber CB, Ollert M (2019). Prevalence of Hymenoptera venom allergy and sensitization in the population-representative German KORA cohort. Allergo J Int.

[27] Hofmann SC, Pfender N, Weckesser S, Huss-Marp J, Jakob T (2011). Added value of IgE detection to rApi m 1 and rVes v 5 in patients with Hymenoptera venom allergy. J Allergy Clin Immunol.

[28] Müller U, Schmid-Grendelmeier P, Hausmann O, Helbling A (2012). IgE to recombinant allergens Api m 1, Ves v 1, and Ves v 5 distinguish double sensitization from crossreaction in venom allergy. Allergy..

[29] Müller UR, Johansen N, Petersen AB, Fromberg-Nielsen J, Haeberli G (2009). Hymenoptera venom allergy: analysis of double positivity to honey bee and Vespula venom by estimation of IgE antibodies to species-specific major allergens Api m1 and Ves v5. Allergy..

[30] Jakob T, Rafei-Shamsabadi D, Spillner E, Müller S (2017). Diagnostics in Hymenoptera venom allergy: current concepts and developments with special focus on molecular allergy diagnostics. Allergo J Int.

[31] • Perez-Riverol A, Palma MS, Jakob T. Current challenges in diagnostics of insect venom allergy. Allergo J Int. 2020. 10.1007/s40629-018-0089-4. **An interesting review on current limitations and future needs in CRD of Hymenoptera venom allergy*****.***

[32] Vos B, Köhler J, Müller S, Stretz E, Rueff F, Jakob T (2013). Spiking venom with rVes v 5 improves sensitivity of IgE detection in patients with allergy to Vespula venom. J Allergy Clin Immunol.

[33] Jakob T, Köhler J, Blank S, Magnusson U, Huss-Marp J, Spillner E, Lidholm J (2012). Comparable IgE reactivity to natural and recombinant Api m 1 in cross-reactive carbohydrate determinant-negative patients with bee venom allergy. J Allergy Clin Immunol.

[34] Korosec P, Valenta R, Mittermann I, Celesnik N, Erzen R, Zidarn M (2011). Low sensitivity of commercially available rApi m 1 for diagnosis of honeybee venom allergy. J Allergy Clin Immunol.

[35] Sturm GJ, Hemmer W, Hawranek T, Lang R, Ollert M, Spillner E, Blank S, Bokanovic D, Aberer W (2011). Detection of IgE to recombinant Api m 1 and rVes v 5 is valuable but not sufficient to distinguish bee from wasp venom allergy. J Allergy Clin Immunol.

[36] Selb J, Bidovec Stojkovic U, Bajrovic N, Kopac P, Erzen R, Zidarn M (2018). Limited ability of recombinant Hymenoptera venom allergens to resolve IgE double sensitization. J Allergy Clin Immunol Pract.

[37] Paulus KE, Spiric J, Junker A, Schwaben L, Lidholm J, Vieths S (2018). Api m 10 can be detected qualitatively by mass spectrometry in all products for allergen immunotherapy for honeybee venom allergy. Allergy..

[38] Spiric J, Paulus KE, Schwaben L, Vieths S, Junker A, Mahler V (2018). Complete compositional analysis of honeybee venom therapeutic products by mass spectrometry. Confirmation of all known Api m allergens in one blow. Allergy.

[39] • Ruiz-Leon B, Navas A, Serrano P, Espinazo M, Labrador-Horrillo M, Monsalve RI, et al. Successful adaptation of bee venom immunotherapy for a monosensitized patient to Api m 10. J Investig Allergol Clin Immunol. 2020. 10.18176/jiaci.0498. **A case report that demonstrates how the knowledge of patients’ sensitization profiles can help to treat a patient in a personalized manner*****.*** 10.18176/jiaci.0498 32101173

[40] Wojak H, Rueff F, Oppel E (2017). Occurrence of a protective effect during therapy with a less purified preparation after prior treatment failure of a bee AIT with a highly purified preparation. Allergo J Int.

[41] Rueff F, Przybilla B, Bilo MB, Müller U, Scheipl F, Seitz MJ (2013). Clinical effectiveness of hymenoptera venom immunotherapy: a prospective observational multicenter study of the European academy of allergology and clinical immunology interest group on insect venom hypersensitivity. PLoS One.

